# Dispute settlement understanding on the use of BOTOX^®^ in chronic migraine

**DOI:** 10.1007/s10194-010-0288-y

**Published:** 2011-01-19

**Authors:** Paolo Martelletti

**Affiliations:** Department of Medical and Molecular Sciences, Regional Referral Headache Centre, School of Health Sciences, Sapienza University of Rome, Sant’Andrea Hospital, Via di Grottarossa 1035, 00189 Rome, Italy

Two important scientists such as Jes Olesen and Peer Tfelt-Hansen have recently lodged a complaint regarding the hypothetical weakness of data presented to the Medicines and Healthcare Products Regulatory Agency for the extension of BOTOX^®^ registration also for chronic migraine (CM) in UK [1]. Besides the institutional reply given by Jennifer Kyne from the above-mentioned agency [2], I believe a discussion should develop around the intricate matter of CM’s classification, especially if such medical condition is considered separately from a rapidly expanding pathology such as medication overuse headache (MOH) [3–6].

MOH constitutes a *plus* of CM and it is hard to think about its appearance not being related to CM itself, unless patients attempt counterproductive stoicisms. Since MOH does not stand alone, it should be at least considered a complication of CM and not just a simple form of secondary headache. However, chronicization process and complication given by MOH are present only in particular CM patient subsets, with a different disease progression not necessarily related to an eventual high/low psychiatric comorbidity [7]. That is what clinical practice teaches us. The presumed weakness of BOTOX^®^ registration data for CM prophylaxis has not hindered registration recently carried out by the Food Drug Administration, with the same purposes. My opinion is that considering the redundancy represented by no less than seven “different” triptans for migraine’s acute treatment, *armamentarium* which was strongly favoured by the very scientific community we represent, a welcome to BOTOX^®^ could sound appropriate. Especially in view of triptan’s misuse often observed during migraine’s chronicization process into CM with MOH’s parallel onset. Such deliberation revolves around the fact that while therapeutic offer for the management of migraine crises enhanced through molecules which are quite alike, in terms of activity [8] and different from one another for what concerns the gene molecular response [9–11], CM prophylaxis gained just one drug, namely topiramate, also coming from a different area that is epilepsy [12, 13].

I already expressed this point of view before any such notion gained ground, hoping that serendipity could bring relief to us and our CM patients [14, 15]. Therapeutic intervention is assessed as excellent or modest according to the large number of positive/negative daily practice results. In our public University Hospital, *off label* BOTOX^®^ injections have been regularly administered to 3.753 certified CM patients from April 2001 to July 2010, for scientific as well as therapeutic purposes (14, Internal Regional Reimbursement Files). Today we cannot deny a chance, although sometimes modest, to CM patients. The usual scientific dialectic crushes against patients’ response, which still represents the focus of both our scientific and clinical research [16].

Following the current debate on such sort of *Dispute Settlement Body*, the next future should lead to in-depth examinations on how BOTOX^®^ acts on CM as well as about when and to which CM subset it results more adequate, in order to invert progression of CM itself [17–20] and consequently reduce appearance of MOH and its disheartening relapses [16].
